# Using Machine Learning Techniques to Predict MACE in Very Young Acute Coronary Syndrome Patients

**DOI:** 10.3390/diagnostics12020422

**Published:** 2022-02-06

**Authors:** Pablo Juan-Salvadores, Cesar Veiga, Víctor Alfonso Jiménez Díaz, Alba Guitián González, Cristina Iglesia Carreño, Cristina Martínez Reglero, José Antonio Baz Alonso, Francisco Caamaño Isorna, Andrés Iñiguez Romo

**Affiliations:** 1 Cardiovascular Research Unit, Cardiology Department, Hospital Alvaro Cunqueiro, University Hospital of Vigo, 36213 Vigo, Spain; pablo.juan@iisgaliciasur.es (P.J.-S.); victor.alfonso.jimenez.diaz@sergas.es (V.A.J.D.); 2 Cardiovascular Research Group, Galicia Sur Health Research Institute (IIS Galicia Sur), SERGAS-UVIGO, 36213 Vigo, Spain; jose.baz.alonso@sergas.es (J.A.B.A.); andres.iniguez.romo@sergas.es (A.I.R.); 3 Interventional Cardiology Unit, Cardiology Department, Hospital Álvaro Cunqueiro, University Hospital of Vigo, 36213 Vigo, Spain; 4 Cardiology Department, Hospital Álvaro Cunqueiro, University Hospital of Vigo, 36213 Vigo, Spain; alba.guitian.gonzalez@sergas.es (A.G.G.); cristina.victoria.iglesia.carreno@sergas.es (C.I.C.); 5 Methodology and Statistics Unit, Galicia Sur Health Research Institute (IIS Galicia Sur), SERGAS-UVIGO, 36213 Vigo, Spain; cristinamreglero@gmail.com; 6 Department of Preventive Medicine, University of Santiago de Compostela, 15705 Santiago de Compostela, Spain; francisco.caamano@usc.es; 7 Consortium for Biomedical Research in Epidemiology and Public Health (CIBER en Epidemiología y Salud Pública-CIBERESP), 15782 Santiago de Compostela, Spain

**Keywords:** machine learning, coronary artery disease, major adverse cardiovascular events, young patient, acute coronary syndrome, coronary angiography, prediction models

## Abstract

Coronary artery disease is a chronic disease with an increased expression in the elderly. However, different studies have shown an increased incidence in young subjects over the last decades. The prediction of major adverse cardiac events (MACE) in very young patients has a significant impact on medical decision-making following coronary angiography and the selection of treatment. Different approaches have been developed to identify patients at a higher risk of adverse outcomes after their coronary anatomy is known. This is a prognostic study of combined data from patients ≤40 years old undergoing coronary angiography (n = 492). We evaluated whether different machine learning (ML) approaches could predict MACE more effectively than traditional statistical methods using logistic regression (LR). Our most effective model for long-term follow-up (60 ± 27 months) was random forest (RF), obtaining an area under the curve (AUC) = 0.79 (95%CI 0.69–0.88), in contrast with LR, obtaining AUC = 0.66 (95%CI 0.53–0.78, *p* = 0.021). At 1-year follow-up, the RF test found AUC 0.80 (95%CI 0.71–0.89) vs. LR 0.50 (95%CI 0.33–0.66, *p* < 0.001). The results of our study support the hypothesis that ML methods can improve both the identification of MACE risk patients and the prediction vs. traditional statistical techniques even in a small sample size. The application of ML techniques to focus the efforts on the detection of MACE in very young patients after coronary angiography could help tailor upfront follow-up strategies in such young patients according to their risk of MACE and to be used for proper assignment of health resources.

## 1. Introduction

Coronary artery disease (CAD) is a very important chronic condition associated with aging and consequently is scarcely present in young people [1]. However, different studies have shown an increased incidence of CAD in young subjects over the last decades [2,3]. Even few, those young patients with CAD mean a significant economic and health care-needs burden for the society, thus becoming chronic patients [4]. Hence, focusing efforts on its proper control will be a priority. It is remarkable that there is not a single definition of what is “young” in the literature; in some works, this means less than 35 years, while other definitions cover a range under 55 [3,5]. The Coronary Artery Risk Development in Young Adults Study (CARDIA) group reported a difference in prevalence of 13.3% vs. 5.5% among patients aged 40–45 years and 33–39 years, respectively [6]. As younger is the threshold definition of young, studies have to deal with the difficulty of enrolling the cohort of patients.

On such patients, the prediction of major adverse cardiac events (MACE) has a significant impact on medical decision making after coronary angiography and the selection of treatment. Several attempts have been made to identify patients at risk of new coronary revascularizations, stroke, repeated myocardial infarction, and mortality after coronary angiography in young patients with highly divergent outcomes [7,8]. Factors such as dyslipidemia, diabetes mellitus, or smoking have been previously shown to be associated with an increased risk of MACE after CAD [9,10,11], in accordance with studies on general population that stated the availability of several laboratory, genetic, and imaging biomarkers for cardiovascular event prediction [12,13]. However, due to limitations, such as the reduced number of clinical variables involved, approaches based on logistic regression (LR) prediction models have had only a limited ability to establish the risk of MACE [14].

The implementation of advanced mathematical algorithms is currently boosting changes in the practice of medicine and promising applications at levels never seen before. In particular, this field, known as artificial intelligence, is progressing significantly in areas such as automated clinical decision making and has the potential to dramatically influence the practice of clinical cardiology. Prediction is probably the most advanced area of machine learning (ML) in the realms of medicine and cardiology [15,16]. The greatest interest lies mainly in knowledge generation through clinical data analysis. In this study, we tested whether different ML approaches could predict MACE more effectively than traditional statistical methods using LR in such very young patients.

## 2. Materials and Methods

### 2.1. Study Population

This is a prognostic study of combined data from patients ≤40 years old undergoing coronary angiography at a tertiary hospital. The information was retrospectively obtained through the specific database of the Interventional Cardiology Unit, disaggregated for any personal data. Patient demographics, comorbidities, angiographic and procedural details, in-hospital events, and post-discharge follow-up were analyzed. All records were reviewed for patients requiring coronary revascularization, including percutaneous coronary intervention (PCI) or coronary artery bypass grafting (CABG), with repeated myocardial infarction, stroke, and mortality.

**Figure 1:**
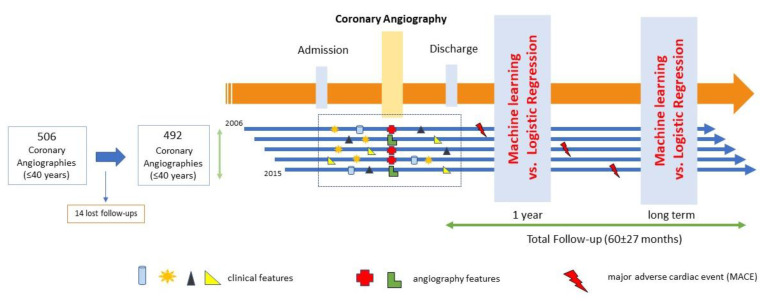
Study flow chart.

All patients ≤40 years old undergoing coronary angiography from 1 January 2006 to 31 December 2015, referred due to clinical suspicion (electrocardiographic changes, biomarkers of myocardial injury, or positive ischemia stress test) of CAD, including acute coronary syndrome (ACS) or stable angina, were included. The exclusion criteria were patients >40 years and referral for coronary angiography for any other non-ischemic cause (pre-transplant, pulmonary hypertension, structural disease, or others). A total of 506 coronary angiograms were performed in this study in young patients during the study period, and during a mean follow-up time of >5 years (the last follow-up was performed on December 2018), 14 patients were lost to follow-up, so they were excluded from the analysis (Figure 1). Each coronary angiography was treated as a separate observation in the study. This study has been approved by the Regional Research Ethics Committee under registration code 2015/506. The whole list of variables is provided in Supplementary Materials as Table S1.

### 2.2. Study Outcomes

The primary study outcome is MACE, a composite endpoint, including all-cause mortality (cardiac and non-cardiac), stroke, repeated myocardial infarction, and coronary revascularization (PCI or CABG). We developed methods to predict two independent outcomes at 1-year follow-up and long-term follow-up.

### 2.3. Machine Learning Model Derivation

Several ML algorithms were independently implemented and configured by the Cardiovascular Research Unit. Continuous data are reported as means with standard deviation. Categorical variables are reported as frequencies and percentages.

Pre-processing of data includes calculating parameters (body mass index from weight and height variables, standardization or mean removal and variance scaling of numerical variables, such as total cholesterol and creatinine, among others) and manually marking each input variable as either numeric or categorical. Missing categorical data were marked as a distinct category. Missing numerical data were completed with the mean if the variable has a normal distribution or with the median if the variable has an asymmetric distribution or just set to zero in cases where the empty variable means no occurrence of event, such as length and diameter of stent.

The registry contained 150 distinct variables, and after dataset dropout, 117 variables were used to perform different algorithms. Variables such as “time to occurrence” were removed from the dataset because they are related to MACE, and they could introduce errors because lower values in this variable mean that the patient has a MACE. Furthermore, variables that were not present in more than 30% of the cases or that were derived from others were excluded from model derivation (n = 32). Target events were identified within the database, and the time from prediction to the event was registered. Data were split in two sets following the standard procedures of ML methodologies and sliced into training (75%) and testing (25%). The MACE variable was used as output label. Based on the input data and labels, several classification technologies were trained and tested. Then, data were used to check the best performance. In this study, six representative supervised ML classifiers, including Support Vector Machine (SVM) [17], Random Forest (RF) [18], Naive Bayes (NB) [19], Multi-layer Perceptron (MLP), Linear Discriminant Analysis (LDA) compared to Logistic Regression (LR) using Lasso (L1), and Ridge Regression (L2) regularization on the regression coefficients [20], were selected and compared to each other due to their proven predictive performance and their popularity in the recently published research literature.

We used several well-known performance measures defined after true-positive, true-negative, false-positive, and false-negative cases obtained with the testing data. The performance metrics to evaluate the learning algorithms include sensitivity, specificity, precision, and accuracy. The receiver operating characteristic (ROC) curves and the area under the ROC curve (AUC) are also provided. This nonparametric method [21], which is commonly used in biomedical research, is employed to compare the models’ curves and investigate the differences between the different ML methods and LR1 (Logistic Regression using Lasso) using the DeLong test, which provides *p*-values and confidence intervals. The ROC curves are considered the standard metric for evaluation of ML models, and we also used the precision/recall (PR) plots due to be more informative when evaluating binary decision problems on imbalanced datasets [22,23]. All these measures were calculated with R 3.6.2 [24] using epiR [25], pROC [26], and PPROC [27] packages.

All classifiers were binary classification models; for each entry on the dataset, there is a non-MACE value 0 or MACE value 1. Performance evaluation was assessed using a K-fold cross-validation procedure (K = 10), a commonly used method to reduce model overfitting by subsetting the available data in k groups for separate analysis. We repeated the learning process ten times to measure the average performance and the 95% confidence interval of each classifier, and 10-fold cross-validation was performed on each (results of such process are provided in Appendix A as Table A1). Such codes were written in python 3.7 using scikit-learn libraries [28]. Algorithms were configured with the following parameters: NB using a maximum number of iterations of 300, RF using 300 estimators, and maximum number of trees of 100, SVM using a linear kernel and *C* equals 1.0, MLP with a = 1, and 150 hidden layers and LDA with standard configuration.

Once the classifiers were trained (fit) using the training dataset, test data were employed to measure the accuracy of each classifier to predict MACE occurrence. The prediction of MACE was used as a score with a receiver-operating characteristic curve drawn and the area underneath it calculated for each target event. As the number of MACEs is limited in the population, the error margins around this curve were determined using a bootstrapping process, a method for determining margins of error by repeated sampling of the data with replacement.

### 2.4. Logistic Regression Model Comparison

In comparison to ML models, the well-established LR method has been employed as the baseline approach to predict MACE in our dataset. The same raw features used for several models were used for the LR internal reference models. Remaining missing values were imputed: numerical features using mean or median training data and categorical variables using the mode of the feature as a global predictor and as a 1-year prediction. LR was configured with L1 and L2 regularization and maximum number of interactions 600.

## 3. Results

During the study period, a total of 19,321 coronary angiograms were performed, 506 of which (2.6%) were in subjects ≤40 years with suspected CAD. The mean age of the population was 35 ± 4.3 years. Chest pain was the most frequent symptom at hospital admission of the patients (87.2%), and the most common presentation was STEMI in 215 patients (43.5%, (Table 1). Coronary angiography showed 340 patients (67.2%) with CAD and 166 (32.8%) without CAD. During the follow-up, the following were detected: 81 (16.5%) repeated coronary revascularizations (PCI or CABG), 14 (2.8%) patients died, 16 (3.3%) had repeated acute myocardial infarction, and 7 (1.4%) had a stroke. A total of 101 (20.5%) MACE were detected in the long-term follow-up.

### 3.1. MACE Prediction Models Long-Term Follow-Up

**Figure 2:**
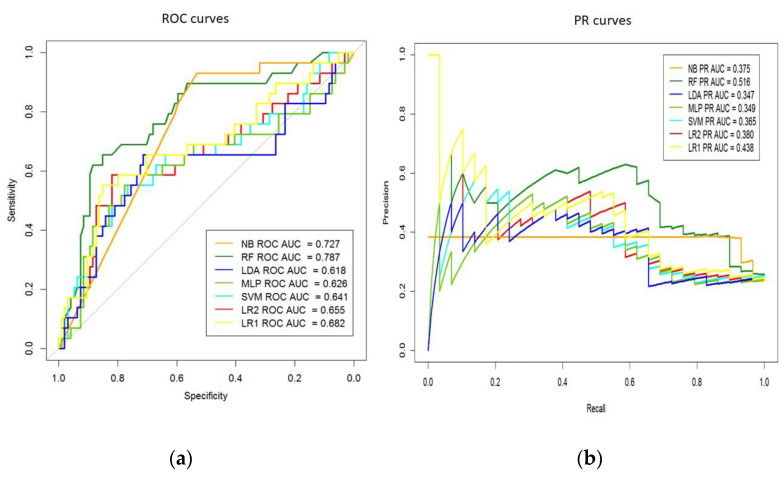
(**a**) Areas under the receiver operating characteristic (ROC) and (**b**) precision/recall (PR) curves for machine-learning models. AUC, Area Under the Curve; LDA, Linear Discriminant Analysis; MLP, Multi-layer Perceptron; NB, Naive Bayes; RF, Random Forest; LR, Logistic Regression; SVM, Support Vector Machine.

To evaluate the performances of the proposed feature extraction methods, we randomly split at 0.75, selecting 123 testing patient samples from the pool of admission records, excluding the 369 records used in the training set. We employed metrics of the AUC-ROC and AUC-PR curves to evaluate the learned models and the baseline models. As shown in Table 2, all four models showed good performances running on the datasets. We computed the above-mentioned performance statistics for each method. RF achieved the best performance with 0.79 AUC-ROC value, with a sensitivity value of 69%, specificity value of 70%, accuracy of 0.70 and precision of 0.42, and a *p*-value of 0.021 obtained by comparison to LR (L2) using DeLong test (Table 2). Additionally, the PR model showed the best result for RF classifier with 0.52 AUC. The AUC-ROC and AUC-PR values for each classifier shown in Figure 2 evidence that the RF approach provides the best accuracy of AUC.

### 3.2. MACE Prediction Models 1-Year Follow-Up

In this case, the output variable to supervise the learning process of the ML classifiers was derived from the “time to occurrence” variable; such variable was not considered in the previous section due to its hidden information of masked events. Now, if an event occurs in less than 365 days, it is set to 1 and to 0 in any other case. The methodology and setting parameters of the classifiers are the same as those described above. The main findings are in terms of classification technologies. The best result is provided by RF but giving the following results in terms of AUC-ROC, sensitivity, specificity, accuracy, and precision, they are 0.80, 75%, 72%, 0.72, and 0.29, respectively. The study reported statistically significant differences for AUC-ROC vs. LR (Table 3).

### 3.3. Extraction of Variables’ Importance and Model Explainability

Once we identified RF as the classifier that provides the best results for this dataset, such classifier was employed to identify the set of most important variables (called features in the ML community) of the dataset related to MACE prediction. This assumes that the input variables have the same scale or have been scaled prior to fitting a model.

**Figure 3:**
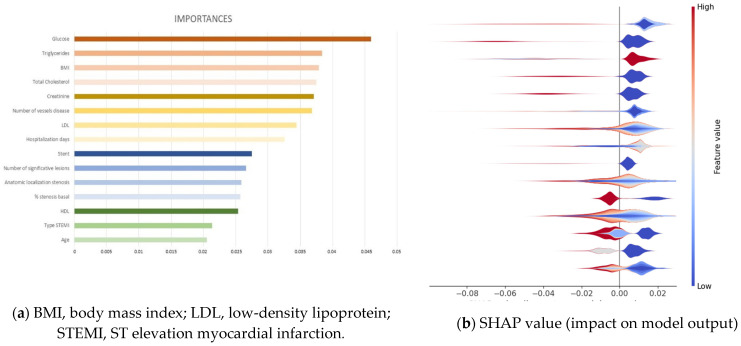
(**a**) Relevance of the 15 most important clinical variables extracted from the Random Forest classifier to long-term follow-up. (**b**) SHAP analysis for those 15 most important clinical variables.

These predictors were identified by evaluating the effect of random permutations of each predictor on the training error of the model using out-of-bag predictor importance estimation [25]. In this way, the study provides a list of variables’ importance that is displayed on Figure 3a, including their relevance for the 15 most important clinical variables extracted, in decreasing order. Such analysis shows that the most important feature is glucose, with a relative importance value of 0.046, and the least important in the list is age, with a relative importance of 0.021. An analysis of the ranked features shows that the variables extracted by RF are quite clinically sound and can effectively reflect the patient’s conditions. Therefore, they are suitable for our prediction task.

Furthermore, explainable machine learning techniques could be employed [29] using SHAP analysis to identify where a SHAP value of features affects the prediction models, namely if a feature affects having MACE or not. Such a SHAP values plot can further show the positive and negative relationships of the variables with the target variable (1 or 0 predictions of the classifier, namely having a MACE or not, respectively), in other words, information about the way a variable is contributing to predict MACE. For each variable, the color shows whether this variable has high values (in red) or low values (in blue) for each observation on the training dataset. The impact is provided by the horizontal location that shows whether the effect of that value is associated with the occurrence of MACE (high, in the right side) or its nonoccurrence (low, in the left side). Figure 3b provides the impact on model output of the 15 most important features, in which it can be observed that the most important variable is glucose, on the top of the plot, where the prediction of MACE (right side of the graph) is associated to samples presenting low values (blue spot). The second variable in importance is triglycerides, showing the same behavior as the first one. The third most important on the list is BMI but with an opposite color; red colors of all samples indicate that high values of this variable contribute to predict MACE. The same analysis can be conducted for all variables of the list; however, the complex interplay of some variables, evidenced by the heterogeneous patterns with mixed colors and locations, would require a longer analysis that is out of the scope of this paper.

## 4. Discussion

In our cohort of young patients undergoing coronary angiography, ML algorithms showed better performance metrics to predict MACE vs. LR prediction models. To the best of our knowledge, this is the first report describing the ML approach for interpreting a data set using hospital admission/discharge and coronary angiographic variables to determine the best method to identify very young patients at risk of MACE.

ML models had good discriminatory ability, especially RF classifier. ML methods outperformed most logistic regression models, resulting in significant net reclassification improvement for MACE at 1-year (*p* < 0.001) and long-term follow-up (*p* = 0.021). There are several potential explanations for the incremental gains in the predictive ability of the ML approaches, as RF approaches can incorporate high-order nonlinear interactions between predictors, which cannot be addressed by logistic regression models. As the dataset is not too large, it was not necessary to apply approaches that minimize overfitting of the model. Several variables were removed, and for several approaches to enhance the validation schemes to solve a small number of MACE cases, cross-validation was implemented using bootstrapping. In addition, our models incorporated all available variables, many of which are unlikely to be included in classical regression models. Preemptive identification of high-risk young patients and conducting a closer clinical follow-up might reduce the incidence of MACE in the long term. Moreover, in the high-risk population, the use of proven strategies, such as optimal medical treatment at hospital discharge for all their CAD risk factors, in addition to an intensive health education might reduce the likelihood of MACE in a resource-efficient manner.

In our study, ML methodologies, specifically the RF approach, show the highest AUC value, which implies a high predictive capacity. This condition will help detect the majority of young patients who, after coronary angiography, are at a high risk of MACE, similar to that reported by other authors [30,31,32]. However, this test had a low sensitivity and precision, maybe due to the reduced sample size and endpoints collected in our study. It would be interesting to analyze prospectively the implementation of these methods for the reduction of MACE, including a larger, multicentric dataset. Only RF showed statistical differences for AUC vs. LR. In addition, the other ML techniques used show an AUC comparable to LR. These results are not sufficient to improve the current MACE-prediction techniques in the young population. In this study, this may be due to the limited number of events not exceeding 20% in the period studied.

At 1-year follow-up, RF continues to be highly valuable for AUC vs. other approaches; the rest of the ML techniques lose performance in terms of AUC, sensitivity, specificity, accuracy, and precision when trying to predict in more restrictive conditions. In this study, we have proposed a 1-year analysis because it is the time when patients visit the physician after hospital discharge. Such loss of performance could be understood in terms of number of MACEs, while at the end of the study, the number of MACEs to be used for the whole process (training and testing) was 101, and the number of MACE at 1 year is 53, which is about a half and represents a small number for extracting clear patterns.

According to these results, it would be interesting that the RF model could be linked with an electronic medical record system to automatically incorporate patient-level data to have an estimated risk score at the time of hospital discharge. This will increase the use of a predictive model in the clinical practice. Therefore, patients identified to be at the highest risk of MACE after coronary angiography complications through these models can be targeted for specific secondary prevention approaches.

The use of ML techniques allows to obtain a list of clinical variables with their relative importance in the RF classifier that could predict MACE. In this study, we give a list of the 15 most significant variables provided by RF, the best classifier obtained. It is interesting to note that classical risk factors, such as LDL, have a lower importance compared to glucose, creatinine, or triglycerides, among others, for predicting MACE. All of this emphasizes the multifactorial nature of the variables that cause MACE after coronary angiography. In addition, half of these variables are cardiovascular risk factors that can be modified applying strict health care strategies; the incidence of MACE during the follow-up could decrease, achieving a positive impact on the health condition of these patients.

The study has several limitations. First, it is a single-center study with a limited number of cases. Second, the number of patients is small in comparison to the number of variables measured per patient. Third, the number of variables measured is limited due to use of a specific database, so many other variables captured in the electronic medical records of the patients were missed for the purpose of this analysis, and their inclusion could improve the training process of the algorithm.

## 5. Conclusions

Our analysis suggests that ML provides a better identification and prediction of MACE in patients undergoing coronary angiography compared with LR even in a small sample size population. The current study promotes the potential role for the integration of ML into the clinical practice for the identification of high-risk young patients after coronary angiography. Applying this ML technique for focusing the efforts to predict MACE in young patients could help physicians make the best decisions based on the risk of MACE. Furthermore, this is a priority given the limited capacity of the health care system to provide personalized management in different countries and the high social-health costs that these events entail for the community.

## Figures and Tables

**Table 1:** Clinical characteristics of patients ≤40 years undergoing coronary angiography.

Variables	Overall (n = 492)
Women	60 (12.2%)
Follow-up time (months)	60 ± 27
Body mass index	28 ± 5
Hypertension	113 (23.0%)
Diabetes mellitus	43 (8.7%)
Smoking	381 (77.4%)
Dyslipidemia	217 (44.1%)
Family history of CAD ^1^	132 (26.8%)
Previous revascularization	62 (12.6%)
Cocaine	52 (10.6%)
Alcohol abuse	52 (10.5%)
Cannabis	56 (11.4%)
Peripheral artery disease	7(1.4%)
Congestive heart failure	3 (0.6%)
Previous stroke	3 (0.6%)
Atrial fibrillation	3 (0.6%)
Renal failure	27 (5.5%)
Depression	44 (8.9%)
Total cholesterol (mg/dL)	194 ± 53
LDL-cholesterol (mg/dL)	124 ± 48
HDL-cholesterol (mg/dL)	39 ± 11
Triglycerides (mg/dL)	162 ± 114
Creatinine (mg/dL)	1.28 ± 1.8
Glucose (mg/dL)	107 ± 44
LVEF ^2^ (%)	55 ± 9
Hospitalization days	6 ± 7

^1^ CAD, coronary artery disease; ^2^ LVEF, left ventricular ejection fraction.

**Table 2:** Prediction Ability of the Reference Model (LR2, Linear Regression2) and five Machine Learning Models, measured in terms of AUC-ROC, Sensitivity, Specificity, Accuracy, and Precision values to predict MACE, using 8-fold cross-validation of 123 patients from the testing dataset.

CLS	AUC (95% CI)	*p*-Value	Sensitivity (95% CI)	Specificity (95% CI)	Accuracy (95% CI)	Precision (95% CI)
Prediction Ability Long-Term Follow-Up						
**LR2**	0.66 (0.53–0.78)	---	0.59 (0.39–0.76)	0.79 (0.69–0.86)	0.74 (0.65–0.81)	0.46 (0.29–0.63)
**NB**	0.73 (0.64–0.81)	0.193	0.97 (0.82–0.99)	0.03 (0.01–0.09)	0.25 (0.18–0.34)	0.23 (0.16–0.32)
**LDA**	0.62 (0.48–0.75)	0.167	0.55 (0.36–0.74)	0.74 (0.64–0.83)	0.70 (0.61–0.78)	0.40 (0.25–0.57)
**RF**	0.79 (0.69–0.88)	0.021	0.69 (0.49–0.85)	0.70 (0.60–0.79)	0.70 (0.61–0.78)	0.42 (0.28–0.57)
**MLP**	0.63 (0.49–0.76)	0.143	0.48 (0.29–0.67)	0.82 (0.73–0.89)	0.74 (0.65–0.81)	0.45 (0.27–0.64)
**SVM**	0.64 (0.51–0.77)	0.689	0.38 (0.21–0.58)	0.85 (0.76–0.92)	0.74 (0.65–0.81)	0.44 (0.24–0.65)
**LR1**	0.68 (0.56–0.80)	0.009	0.59 (0.39–0.76)	0.80 (0.70–0.87)	0.74 (0.66–0.82)	0.47 (0.30–0.64)

CLS, classifiers; AUC, Area Under the Curve; CI, confidence interval; LR, Logistic Regression; NB, Naive Bayes; LDA, Linear Discriminant Analysis; RF, Random Forest; MLP, Multi-layer Perceptron; SVM, Support Vector Machine.

**Table 3:** Prediction Ability of the Reference Model (LR2, Linear Regression2) and five Machine Learning Models, measured in terms of AUC-ROC, Sensitivity, Specificity, Accuracy and Precision values to predict MACE, using 8-fold cross-validation of 123 patients from the testing dataset.

CLS	AUC (95% CI)	*p*-Value	Sensitivity (95% CI)	Specificity (95% CI)	Accuracy (95% CI)	Precision (95% CI)
Prediction Ability One-Year Follow-Up						
**LR2**	0.50 (0.33–0.66)	---	0.25 (0.073–0.52)	0.81 (0.73–0.88)	0.74 (0.65–0.81)	0.17 (0.05–0.38)
**NB**	0.47 (0.33–0.60)	0.741	0.75 (0.48–0.93)	0.06 (0.02–0.12)	0.15 (0.09–0.22)	0.11 (0.06–0.18)
**LDA**	0.49 (0.31–0.67)	0.970	0.37 (0.15–0.66)	0.78 (0.69–0.86)	0.73 (0.64–0.80)	0.21 (0.08–0.40)
**RF**	0.80 (0.71–0.89)	<0.001	0.75 (0.48–0.93)	0.72 (0.62–0.80)	0.72 (0.64–0.80)	0.29 (0.16–0.45)
**MLP**	0.56 (0.40–0.71)	0.159	0.25 (0.07–0.52)	0.84 (0.75–0.90)	0.76 (0.68–0.84)	0.19 (0.05–0.42)
**SVM**	0.45 (0.28–0.62)	0.271	0.06 (0.00–0.30)	0.87 (0.79–0.93)	0.76 (0.68–0.84)	0.07 (0.00–0.32)
**LR1**	0.61 (0.45–0.78)	0.066	0.56 (0.30–0.80)	0.52 (0.42–0.62)	0.53 (0.44–0.62)	0.15 (0.07–0.27)

CLS, classifiers; AUC, Area Under the Curve; CI, confidence interval; LR, Logistic Regression; NB, Naive Bayes; LDA, Linear Discriminant Analysis; RF, Random Forest; MLP, Multi-layer Perceptron; SVM, Support Vector Machine.

## Data Availability

The data used to support the findings of this study are available from the corresponding author upon reasonable request.

## Supplementary Materials

The following supporting information can be downloaded at: https://www.mdpi.com/article/10.3390/diagnostics12020422/s1, Table S1, The whole list of variables.

## Appendix A

**Table A1:** Results of the K-fold cross-validation.

	LR2	NB	LDA	RF	MLP	SVM	LR1
**K = 0**	0.91	0.39	0.73	0.82	0.91	0.91	0.82
**K = 1**	0.86	0.55	0.82	0.82	0.82	0.77	0.77
**K = 2**	0.77	0.32	0.73	0.90	0.80	0.70	0.90
**K = 3**	1.00	0.65	0.50	0.95	0.90	0.95	1.00
**K = 4**	0.95	1.00	0.95	0.90	0.90	1.00	0.95
**K = 5**	0.70	0.20	0.80	0.50	0.65	0.70	0.75
**K = 6**	0.95	0.95	0.50	0.95	1.00	0.85	1.00
**K = 7**	0.25	0.00	0.00	0.80	0.55	0.20	0.70
**K = 8**	0.80	0.35	0.65	0.70	0.70	0.75	0.75
**K = 9**	0.85	0.75	0.73	0.55	0.90	0.95	0.80

NB: Naive Bayes. LDA: Linear discriminant analysis. RF: Random forest. MLP: Multi-layer Perceptron. LR: Logistic regression using Lasso (LR1) and Ridge Regression (LR2). SVM: Support Vector Machine.
