# Supplementary material for: Using Machine Learning Techniques to Predict MACE in Very Young Acute Coronary Syndrome Patients
Source: Diagnostics (Basel). 2022 Feb 6;12(2):422. doi: 10.3390/diagnostics12020422 (PMC8870965; doi:10.3390/diagnostics12020422)
Supplement: Supplementary file 1 [file diagnostics-12-00422-s001.zip › diagnostics-1550945-supplementary.pdf]

Supplementary Materials. Table S1, The whole list of variables

1. Cases and control
2. Percutaneous coronary intervention (PCI) day
3. Data of hospital discharge
4. Number of PCI
5. Previous revascularization
6. Sex
7. Hypertension
8. Diabetes Mellitus
9. Smoking
10. Dyslipidaemia
11. Family History of Coronary Disease
12. Peripheral Vascular Disease
13. Type of Previous revascularization
14. Previous myocardial infarction
15. PREVIOUS Congestive Heart Failure
16. PREVIOUS Percutaneous Revascularization
17. Type of heart attack
18. PRIOR Surgical Revascularization
19. PREVIOUS stroke
20. RISK FACTORS NEW DIAGNOSIS
21. Atrial Fibrillation
22. Renal Insufficiency
23. Depression
24. Cannabis
25. Heroin
26. Alcohol
27. Cocaine
28. Antiplatelet therapy (not ASA) Pre-PCI
29. Acetyl Salicylic Acid Pre-PCI
30. Pre-PCI Anticoagulants
31. Pre-PCI betablockers
32. Pre-PCI Angiotensin-converting-enzyme inhibitors
33. PCI Angiotensin II receptor blockers
34. Pre-PCI Calcium Channel Blockers
35. Pre-PCI Statins
36. Pre-PCI Diuretics
37. Pre-PCI Oral Antidiabetics
38. Pre-PCI Antiarrhythmics
39. Chest pain Symptoms on admission
40. Dyspnoea Symptoms on Admission
41. Syncope Symptoms on Admission
42. Anatomical location of the AMI
43. Total Cholesterol
44. HDL cholesterol
45. LDL cholesterol

46. Triglycerides
47. Creatinine
48. Glucose
49. Indication for catheterization
50. Angioplasty modality
51. Discharge diagnosis
52. Type of complete or incomplete revascularization
53. Observed Left Ventricular Ejection Fraction (%)
54. LVEF coded dichotomous good / bad (40 or less poorly preserved)
55. Date of hospital admission
56. Hospitalization nights
57. IP2Y12 at hospital discharge
58. ASA at discharge from hospital
59. Anticoagulants at hospital discharge
60. Beta-blockers at hospital discharge
61. Angiotensin-converting-enzyme inhibitors at Hospital Discharge
62. PCI Angiotensin II receptor blockers at Hospital Discharge
63. Calcium channel blockers at hospital discharge
64. Statins at hospital discharge
65. Diuretics at hospital discharge
66. Oral Contraceptives at hospital discharge
67. Oral antidiabetic drugs at hospital discharge
68. Antiarrhythmics at hospital discharge
69. Time from PCI to date of follow-up in months
70. Date when the follow-up was done
71. New coronary revascularization during follow-up
72. Date of the RE-PCI in the follow-up or end of follow-up
73. all revascularization of the treated lesion
74. entire revascularization of the affected vessel
75. all non-revascularization of the affected vessel
76. Death of the patient
77. Cause of death
78. Exitus date or end of follow-up
79. Acute Myocardial Infarction during follow-up
80. AMI date or end of follow-up
81. Cerebrovascular accident in the follow-up
82. Date of stroke or end of follow-up
83. Date of MACE or end of follow-up
84. Combination of events YES / NO
85. Coronary Artery Status
86. Number of diseased vessels
87. Number of lesions treated
88. Number of vessels treated
89. Number of severe injuries> 75%
90. Number of non-severe injuries <75%
91. Number of stents per procedure

92. Coronary artery where the main lesion is located
93. % of basal stenosis
94. Basal TImi Flow Classification
95. AHA classification of the type of coronary lesion
96. The lesion presents calcium
97. Lesion presents thrombus
98. Lesion located in a bifurcation
99. Type of treatment stent, balloon, thrombus aspiration, referred to surgery
100. 100% final stenosis
101. PCI Final TImi Flow Classification
102. Result of the treatment SUCCESS / FAILURE
103. Reason for a non-PCI
104. Thrombus aspiration has been performed
105. It has been possible to extract thrombus
106. Diameter of implanted stent
107. Length of implanted stent
108. Trade name of the implanted stent
109. Type of drug covers stent or conventional
110. Coronary artery where the 2 lesion treated in the same procedure is located
111. % of basal stenosis lesion 2
112. Baseline TIMI flow classification lesion 2
113. Classification Flow TIMI final injury 2
114. Diameter of implanted stent lesion 2
115. Length of the implanted stent lesion 2
116. Trade name implanted stent lesion 2
117. Type of drug eluding stent or Bare Metal Stent
